# Relationship between Vehicle Safety Ratings and Drivers’ Injury Severity in the Context of Gender Disparity

**DOI:** 10.3390/ijerph19105885

**Published:** 2022-05-12

**Authors:** Wen Fu, Jaeyoung Lee

**Affiliations:** 1 School of Traffic & Transportation Engineering, Central South University, Changsha 410075, China; fuwenw@csu.edu.cn; 2 Department of Civil, Environmental & Construction Engineering, University of Central Florida, Orlando, FL 32816, USA

**Keywords:** traffic safety, gender disparity, vehicle safety rating, random parameters model, heterogeneity

## Abstract

Previous studies have analyzed the relationship between vehicle safety ratings from impact tests and actual crash injury severity. Nevertheless, no study has investigated the relationship in the context of gender disparity. The main objective of this paper is to explore the validity of the 5-star ratings of the U.S. National Highway Traffic Safety Administration, which describes vehicles’ protectiveness, using actual traffic crash data by gender. Random parameter models are developed using 2015–2020 two-vehicle crash data from Maryland, United States. According to the data, over 90% of vehicles have 4–5 stars in overall, front-impact, and side-impact 5-star ratings. After controlling other factors, it is shown that woman drivers are more likely to be seriously injured in two-vehicle crashes than men drivers when using vehicles with the same 5-star safety ratings. Moreover, there is significant individual heterogeneity in the effect of vehicles with different 5-star safety ratings on driver injury severity. Using vehicles with more stars can reduce the risk of being seriously injured for most man drivers. However, the probability of woman drivers being seriously injured is reduced by approximately 5% on average by using vehicles with higher star ratings in the overall and front-impact 5-star rating, and individual heterogeneity shows a difference of nearly 50% in positive and negative effects. The overall and front-impact 5-star ratings of vehicles could not provide reasonable information as the safety performance of vehicles in traffic crashes for woman drivers. On the other hand, drivers’ residence, driving characteristics, crash types, and environmental characteristics are significantly associated with the injury severity. It is expected that the results from this study will contribute to guide a better vehicle safety design for both men and women.

## 1. Introduction

In 1978, the U.S. National Highway Traffic Safety Administration (NHTSA) began rating the protectiveness of vehicles for drivers based on front-impact test, and the NHTSA has conducted a side-impact test since 1996 and a rollover-crashes test since 2000 [1]. All of tests are called 5-star safety rating project, which divided impact safety of vehicles into 5 levels: from 1-star to 5stars. Vehicle with more stars indicates that it is more capable to protect drivers and occupants from being injured or fatal in traffic crashes. The 5-star safety ratings of vehicles could encourage customers to purchase safer vehicles, and could promote manufacturers to make safer vehicles that can gain more stars. Finally, the government can utilize the project of vehicle safety rating to realize safer traffic system by the decreasing of the injury severity or fatality risk in traffic crashes. Besides the 5-star safety rating project, the Insurance Institute for Highway Safety (IIHS), vehicle manufacturers (e.g., Volvo), New Car Assessment Programs (NCAP) of many countries developed their vehicle safety ratings based on the reference of 5-star safety rating. Researchers have been interested in identifying whether those safety ratings of vehicles effectively reflect the vehicles’ actual protectiveness in traffic crashes. Lie and Tingvall studied the correlation between the European NCAP ratings of vehicles and drivers’ injury risks in two-vehicle crashes. The authors found that a lower-rating vehicle is related to severe injury risks [2]. Braver and Kyrychenko found that the safety rating of Volvo vehicles’ side impact protection is trustworthy and made a conclusion that the newer protection system can better protect occupants [3]. Teoh and Lund analyzed the relationship between side-impact ratings of vehicles from the IIHS and occupants’ death risk in traffic crashes. They found that the highly-rated vehicles can significantly reduce occupant fatalities [4].

Meanwhile, a gender difference in traffic safety has been reported by multiple studies [5,6,7]. Obeng found that women’s injury severity risks are higher than men’ when considering driver’s conditions such as impairment, illness, drowsiness [8]. Dasari et al., also found gender differences in the risk of death after trauma from traffic crashes [9]. Furthermore, the NHTSA began to consider the gender disparity in 5-star safety rating project in 2003 by adding a woman dummy in front-impact safety test, then adding a woman dummy in side-impact safety test in 2006. Fu et al., found the countermeasure adding a woman dummy in 5-star safety rating could effectively promote the safety design of vehicles for drivers by gender in traffic crashes, but the safety performance of vehicles still differs for man and woman drivers [10]. Importantly, Bose et al., found that the man-oriented design of testing dummies in vehicle safety rating project made vehicles provide the inequitable protectiveness for drivers by gender, and they proposed gender-specific effectiveness of vehicle impact safety is dependent on anthropometric measures, injury tolerance, and associate biomechanical response [11]. Therefore, it is questionable that the vehicle safety rating is effective for providing reasonable information to customers and vehicle manufacturers about vehicle impact safety for drivers or occupants by gender.

To the author’s best knowledge, no researcher has explored the relationship between vehicle safety ratings and drivers’ injury severity in the context of gender disparity. Therefore, the main objective of this paper is to explore the validity of the NHTSA’s 5-star safety ratings of vehicles for drivers to illustrate the safety performance of vehicles in the perspective of gender equality using actual crash data.

## 2. Literature Review

### 2.1. Relationship between Vehicle Safety Rating and Real-World Crashes

Many research studies have focused on the relationship between vehicle safety rating and real-world traffic crashes, especially evaluating the validity of the vehicle safety ratings based on analysis of traffic crash data. Hackney utilized the 1978–1992 fatal crash data to explore the safety performance of vehicles with different ratings from the NCAP by analysis of head-on collisions. The authors found highly-rated vehicles are associated with a lower risk of fatality for drivers [12]. Lie and Tingvall studied the correlation between European NCAP’s results and passengers’ injuries in two-vehicle crashes. The authors found significant injury risk differences for severe or fatal injuries in vehicles with different ratings, while there was no significant difference for minor injuries [2]. Moreover, Strandroth et al., found that reduction of injury severity of pedestrians in traffic crashes was significantly associated with vehicles with higher pedestrian safety ratings from the European NCAP. The validity of improved policies about vehicle safety rating programs could be evident by modeling actual crash data [13]. Kahane found that the improvement of Thoracic Trauma Index in the Federal Motor Vehicle Safety Standard 214 showed a lower risk of fatality for near-side occupants [14]. Philips et al., found that vehicles with higher ratings from the 5-star safety rating and the IIHS are significantly associated with the reduction of injury severity of occupants, but the effects of higher safety ratings on the injury severity reduction were much less than the authors’ expectation [15].

On the other hand, the analysis of relationship between vehicle safety rating and actual crashes could utilize as the scientific reference to further improve vehicle safety rating programs. Tencer et al., identified vehicle design factors affecting pelvic and thoracic force for drivers in near-side impact crashes and found the door velocity during impact and a side airbag significantly affect occupants’ chest injury, while pelvic acceleration can be reduced by reducing door crush, decreasing wheelbase [16]. Metzger et al., focused on the injury risk of restrained rear-seat passengers in different ratings based on the NCAP of vehicles in real-world crashes. They found that the vehicle impact ratings for driver and passenger were not significantly associated with the injury risk of rear-seat passengers, except for the front-impact ratings of vehicles. It implies that the side-impact rating program should be improved for rear-seat occupants in the future [17]. Moreover, Fu et al., focused on the optimization of dummies in vehicle impact tests of 5-star safety rating. Their results showed that newer vehicles are safer for man and woman drivers by modeling the drivers’ injury severity in real-world crashes, which illustrated that the optimization of dummies are validity to guide the safer design of vehicles [10].

### 2.2. Modeling Injury Severity in Traffic Crashes

Discrete outcome models have been popularly used in modeling injury severity in traffic crashes. Binary/multinomial logit/probit model and ordered logit/probit model have been widely used. Islam et al., and Ulfarsson et al., respectively explored the influence of driver age and vehicle type on drivers’ injury severity by using multiple Logit models [18,19]. Obeng used the ordered Logit regression model to analyze drivers’ injury severity and carried out a quantitative study on gender differences in traffic accidents [8]. Abdel-Aty used the ordered Probit model to explore the drivers’ injury severity in traffic crashes occurring at intersection, land and toll plazas. Their results showed the importance of driver’s age, gender, seat belt use, collision point and other factors on the injury severity [20].

However, the heterogeneity of unobserved factors, independence of irrelevant alternatives, correlation of error terms and other limitations from the abovementioned models all could result in a biased estimation. Therefore, nested logit model [21,22], random parameter model [23], random effect model [24], latent class model [25], multivariate logit/probit model [26] and other mixed models were utilized to eliminate different limitations so that realized the more accurate estimation. Russo et al., proposed a random parameter ordered model for the analysis of passengers’ injury severity, which took into account the potential correlation of passengers in a traffic crash and the unobserved heterogeneity [27]. Kim et al., used the mixed Logit model to analyze the drivers’ injury severity in single-vehicle crashes and found that age and gender led to important heterogeneity effects [28]. In order to capture the heterogeneity effect across categories of traffic crashes, Li et al., proposed a latent classification model, which classifies the entire research data by maximizing the homogenization effect of each cluster, and then conducts modeling drivers’ injury severity for each cluster group separately [29]. Although the traditional discrete outcome model can be improved like abovementioned studies, the choice of models should not only pursue the complexity of model construction and high precision of estimation, but also consider the computational complexity of model and the applicability of actual data. Additionally, if only to predict the injury severity not to focus on a specific factor affecting injury severity, data mining techniques have better performance than statistical models, but those have the limitations of overfitting and non-estimated parameter so that quantitative analysis of the factors is not possible [30,31,32].

In conclusion, this paper was going to focus on relationship between vehicle safety rating from 5-star rating program and drivers’ injury severity in real-world traffic crashes under the context of gender disparity. In addition, this paper also was interested in heterogeneity, and used random parameter model with the heterogeneity of mean to analyze the existence and source of heterogeneity.

## 3. Materials and Methods

### 3.1. Materials

This paper used the crash report data from Maryland from 2015–2020, which including the information of crashes, vehicles and person involving traffic crashes. Besides, vehicles’ 5-star safety ratings were collected from the NHTSA-the file of Safer car. A vehicle make/model/model year could be utilized to uniquely identify its 5-star ratings. Therefore, this paper made the drivers as a unit to integrate data set. Those records that lost the details of drivers’ gender and vehicle’s 5-star ratings were filtered. Moreover, there were several criteria to filter the data.

(1) Vehicles’ safety levels from overall, front-impact and side-impact 5-star ratings

The NHTSA’s 5-star rating project includes four aspects, respectively overall, front-impact, side-impact and rollover ratings. Due to the complexity of the rollover-crash scenario, the records of rollover crashes are not included in this study. According to the statistics of crash frequency, the number of vehicles with 4 stars or above from overall, front-impact and side-impact rating are account for more than 90% of all drivers’ crash records, and there is no vehicle rated 1 star based on 5-star ratings. The above unbalanced data can easily lead to biased estimates if the five safety levels of vehicles are discussed separately. Therefore, the vehicles with 3 stars or below are as the same category in this paper.

(2) Passenger cars manufactured in 2011–2020 in two-vehicle crashes

To avoid a significantly different safety performance from different vehicle types [30,33], this paper analyzed drivers’ injury severity only involved passenger cars. Furthermore, an older vehicle model has a lower level of protection for occupants [10]. Thus, vehicles of model year only in 2011–2020 were used in this study. Thus, vehicles’ model age are 8 years or younger. Additionally, two-vehicle and single-vehicle crashes are account for 82% of all crashes in the data set. However, there is a lack of factors affecting the injury severity considering different conditions of single-vehicle crashes. Single-vehicle crashes include pedestrian-vehicle impact, run-off road crashes and hitting a stationary object, which should be considered when modeling injury severity [34,35]. Therefore, only two-vehicle crashes were analyzed in this study.

(3) Control factors

Multiple significant factors related to driver, vehicle, and crash characteristics were utilized as the control variables for this study. In summary, there are 14,762 drivers’ observations from 7381 two-vehicle crashes in the study. In conclusion, there are some binary variables used in this study. For example, they include “woman driver”, “older driver” and so on. If the variable is encoded as ‘1’, which indicates the driver or the crash has this characteristic, otherwise encoded as ‘0’. Detailed descriptive statistics of factors are shown in Table 1.

Since there is a very small number of incapacitating and fatal injuries for drivers in two-vehicle crashes from the dataset. If the five injury severities of drivers are discussed separately could easily lead to biased estimates. Therefore, this paper decided to combine all severe injuries into one category (i.e., fatal, incapacitating, and possible incapacitating injuries), named: severe injury. In this study, we have a binary dependent variable: 1 if the driver is severe injury and 0 otherwise.

In order to explore the validity of front-impact and side-impact 5-star ratings for vehicles, different impact location of vehicle in traffic crashes were divided. According to the impact locations of vehicles in two-vehicle crash data, there are 9041 drivers’ records involved front-impact crashes and 763 drivers’ records involved driver-side-impact crashes. Control factors would be not repeated here. Key variables’ detailed descriptive statistics are shown in Table 2.

### 3.2. Methods

Random parameter model has been frequently used to consider the observation-oriented heterogeneity, which assumes the variation of estimated parameters across observations. However, the random parameter model only focuses on the existence of heterogeneity across observations, not further considers the source. Therefore, a random parameter model with heterogeneity in mean was applied in this paper.

To estimate the model, a linear function that $U_{i}^{*}$ determines the probability $P_{i}$($Y_{i}=1$) of ith driver’s severe injury ($Y_{i}=1$) according to all the observed variables:

$$U_{i}^{*}=\beta_{0}+\sum_{K}\beta_{k}X_{\mathrm{ki}}+\varepsilon_{i}$$

where $X_{\mathrm{ki}}\;\left(k=1,2,\cdots K;i=1,2,\cdots N\right)$ is the vector of K observed explanatory variables, $\beta_{k}\;\left(k=1,2,\cdots K\right)$ is the vector of estimated parameters, and $\varepsilon_{i}$ represents the disturbance term following a logit distribution. Moreover, the value $U_{i}$ for the ith driver is defined as follows:

$$U_{i}=\ln\frac{P_{i}\left(Y_{i}=1\right)}{1-P_{\begin{array}{c} i \end{array}}\left(Y_{i}=1\right)}=\beta_{0}+\sum_{K}\beta_{k}X_{\mathrm{ki}}$$

Then $P_{i}\left(Y_{i}^{*}=1\right)$ the probability of severe injury for ith driver could be shown as follows:

$$P_{i}\left(Y_{i}^{*}=1\right)=\frac{\mathrm{EXP}\left[\beta_{0}+\sum_{K}\beta_{k}X_{\mathrm{ki}}\right]}{1+\mathrm{EXP}\left[\beta_{0}+\sum_{K}\beta_{k}X_{\mathrm{ki}}\right]}$$

Furthermore, the random parameter mode allows the variability of the coefficient $\beta_{k}$ across observations, and the variability is denoted by the following equation.

$$\beta_{k,i}=\beta_{k}+\gamma_{k,i}\;\;\beta_{0,i}=\beta_{0}+\gamma_{0,i}$$

where,

$\beta_{k,i}$ = coefficient of $X_{k}$ for the ith observation

$\beta_{0,i}$ = random constant for ith observation

$\gamma_{k,i}{\;\mathrm{and}\;\gamma }_{0,i}$= normal-distributed error term

Moreover, random effect was utilized to consider heterogeneity in panel data of different two-vehicle crashes in this study. Significant random constant could illustrate the reasonability of considering random effect. If $\beta_{0,i}$ is significantly shows the random distribution in this study, it is illustrated that the inter-group heterogeneity significantly exists between two-vehicle crashes.

In order to explore the source of heterogeneity across observations, the random parameter model with heterogeneity in mean was utilized. $E\left(\beta_{k,i}\right)$ represents the mean of the random variability of coefficient $\beta_{k}$ across observations, then the random parameter considering the heterogeneity in mean is denoted by the following equation:

$$\left(\beta_{k,i}|X\prime \right)=E\left(\beta_{k,i}\right)+\;\tau \left(X\prime \right)$$

where X′ represents the vector of variables affecting the mean of the random parameter; τ(X′) represents the parameter, which is the change in the mean.

The maximum likelihood approach with 100 Halton draws is employed to estimate the random parameter model with heterogeneity in mean [36,37]. It should be pointed out that different Halton draws were tested to arrive at the most accurate and efficient estimation. Additionally, the study hypothesized the normal distribution for random parameters after comparative analysis, although the other distributions, such as lognormal and uniform etc., can also be used.

On the other hand, the estimated parameters in abovementioned model could be used to show the variables when value is 1 are positively (β>0) or negatively (β<0) associated with the probability of severe injury for drivers in traffic crashes, which is not quantitative interpretation and difficult to understand. Therefore, odds ratio (OR) is utilized in the study to quantified explain the effect of the variables on injury severity of drivers. For example, the estimated parameter of the binary variable $X_{1}$: “Woman driver” is $\beta_{1}$ and then the OR is $\mathrm{EXP}\left(\beta_{1}\right)$ which illustrates that the probability of severe injury for woman drivers ($X_{1}=1$) in traffic crashes is $\mathrm{EXP}\left(\beta_{1}\right)$ times that of man drivers ($X_{1}=0$).

## 4. Results

### 4.1. Considering Overall 5-Star Safety Ratings

A random parameter model with heterogeneity in mean was developed to identify the relationship between overall 5-star safety ratings of vehicles and drivers’ injury severity by gender. The results are shown in Table 3.

According to the results, several control factors are found significantly associated with drivers’ injury risk when two-vehicle crashes had occurred. Drivers who are older than 60 years old are higher likely to be severe injury in traffic crashes than younger drivers, which could be explained by drivers’ health for young drivers [38]. The opposite driver who is at fault in two-vehicle crashes makes the drivers have higher probability of being severe injury, which implied that violation is dangerous behavior for others in traffic system and it could lead to severe crashes [39]. Vehicles with the impact locations of front or driver-side point show 1.188 and 1.428 times higher probability of being severely injured for drivers in two-vehicle crashes, respectively than other impact locations, which is consistent with previous studies [40,41]. Dark without light and a signalized control show significantly associated with the increased probability of being severe injury for all of drivers [42]. Speed limit of land is significantly associated with the risk of severe injury for drivers. On a land with speed limit from 25 to 55 mph, drivers have higher probability of severe injury in traffic crashes than whom on a land with lower speed limit. The similar result is consistent with previous research studies [35,43]. Furthermore, Driver who is changing driving speed on land has 22.6% lower probability of being severe injury in traffic crashes than who is keep constant driving speed. Drivers in vehicles with over 6-years model show 17.6% less likely to be severe injury, which implied that relatively new vehicles’ model do not show additional safety benefits for drivers, and vehicle safety performance has not been improved. Raining, snowing, sleeting and mixing are negatively associated with drivers’ severe injury risk, which implies that complex weather could make drivers drive slowly and carefully [44,45].

Moreover, several variables were found to have random parameters following normal distribution across observations, which show the existence of unobserved heterogeneity. The estimated parameter of the “Constant” follows a normal distribution with a mean of −3.653 and a standard variance of 1.969, which indicated that the inter-crashes has significantly random effect, and there is heterogeneity between different two-vehicle crashes. Additionally, driver from Maryland is random parameter following the normal distribution with a mean of 0.264 and a standard variance of 0.508. The result illustrates that in-state drivers have an average higher probability of being severe injury in two-vehicle crashes, but 30.2% of in-state drivers are negatively associated with severe injury in two-vehicle crashes. Speed limit over 60 mph also shows randomly effect on drivers’ injury severity comparing to the land with speed limit lower 25 mph. Especially, the random distribution mean of the estimated parameters of “Signal control” is found to be affected by “intersection”, and its mean value would increase by 0.630 with the crashes involving an intersection. This result indicates that the heterogeneous influence of signal-controlled facilities on driver injury severity is related to whether an intersection is involved [10,42].

### 4.2. Considering Front-Impact and Side-Impact 5-Star Safety Ratings

Two random parameter models were developed to identify the relationship between front-impact and side-impact 5-star ratings of vehicles and drivers’ injury severity by gender. The results are shown in Table 4. Significant control factors could be reasonably explained by previous research studies by the value: odds ratio, the results about the control factors would be repeat here.

## 5. Discussion

Three random parameter models were developed to explore the validity of 5-star safety ratings for drivers by gender in illustrating the safety performance of vehicles in traffic crashes based on the data from 2015–2020 two-vehicle crashes in Maryland. The results from three models show the significantly correlation between the overall, front-impact and side-impact 5-star ratings of vehicles and the risk of being severe injury for drivers by gender.

According to the Table 3, vehicles with 5 stars and 4 stars from overall 5-star rating show the random effect on drivers’ injury severity in two-vehicle crashes, which illustrated that vehicles with same safety level have heterogeneous effect on drivers’ injury severity across observations. Furthermore, the interaction terms between vehicles with 4 or 5 stars from overall 5-star rating and drivers’ gender are significantly associated with the probability of severe injury for drivers in two-vehicle crashes. The results show that the estimated parameters of vehicles with overall 4 or 5 stars on the probability of severe injury of drivers could positively increase, respectively by 0.579, 0.390, when the driver is woman.

**Figure 1:**
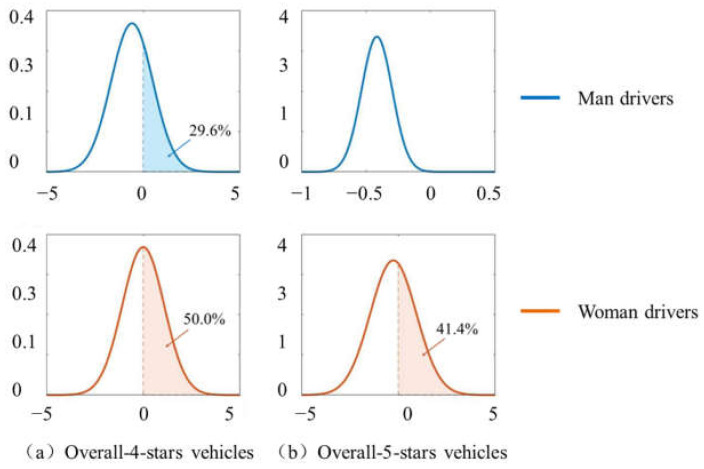
Random distribution of the parameter “Overall 5-star ratings of vehicles”.

For man drivers, the probability of severe injury in vehicles with overall 4 or 5 stars are averagely 44.0% and 34.0% lower than whom in vehicles with overall 3 or less star. On average, vehicles with overall 4 stars show a safer performance for the risk of drivers’ severe injury than vehicles with overall 5 stars. However, considering the distribution of random parameters, about 70.4% of man drivers reduce their probability of severe injury by using vehicles with overall 4 stars in traffic crashes, while nearly 100% of man drivers reduce their probability of severe injury by using vehicles with overall 5 stars. The results indicate that overall-5-stars vehicles do not averagely further reduce the probability of severe injury for all of man drivers, but it contains more heterogeneous effects than overall-4-stars vehicles so that enable a wider range of man drivers to obtain better safety performance from overall-5-stars vehicles. The overall 5-star ratings of vehicles could provide reasonable information as the safety performance of vehicles in traffic crashes for man drivers, to some extent, which is similarly consistent with some previous research studies without considering gender differences [2,4,13]. For woman drivers, vehicles with overall 4 or 5 stars are averagely negatively associated with the probability of severe injury. The random effect of vehicles with overall 4 or 5 stars across drivers on the probability of severe injury in two-vehicle crashes is shown in Figure 1. The mean of random distribution of the parameters of vehicles with overall 4 and 5 stars for woman is close to 0, indicating that there is no significant difference, on average, in the probability of severe injury in two-vehicle crashes for woman drivers using vehicle with higher 5-star ratings. About 50 percent of woman drivers do not receive the additional safety protection they deserve from vehicles with overall 4 or 5 stars, compared with vehicles with 3 and less stars. Vehicles with overall 4 or 5 stars do not show better protection performance to woman drivers, and the heterogeneity significant affect on the injury severity. The overall 5-star ratings of vehicles could not provide woman drivers with reasonable information about vehicle impact safety.

Furthermore, woman drivers are 78.4% more likely than man drivers to be severe injury when driving overall-4-stars vehicles, and 47.7% more likely when driving overall-5-stars vehicles comparing to whom driving vehicles with overall 3 or less stars. The results indicate that there are significant differences for man and woman drivers in the protection performance of vehicles with the same overall 5-star ratings, and man drivers are in a relatively safer protection of vehicles when involving traffic crashes. Some researchers also found this result [46,47].

**Figure 2:**
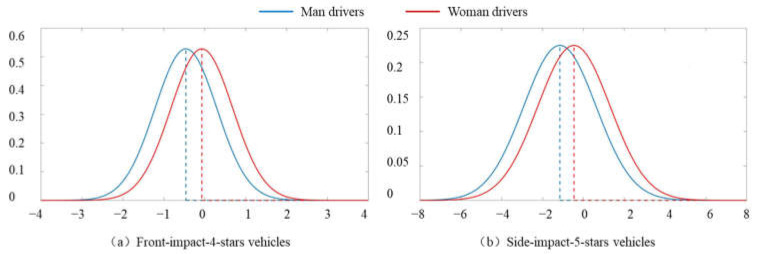
Random distribution of the parameter different 5-star ratings of vehicles.

Modeling relationships between front-impact and side-impact 5-star ratings of vehicles and drivers’ injury severity in front-impact and driver-side-impact crashes, the results (Table 4) show that there is significantly heterogeneity across observations when considering vehicles with same safety level from corresponding 5-star rating. Moreover, the interaction terms between vehicle with different 5-star ratings and drivers’ gender are found significantly related to the probability of severe injury for drivers. The abovementioned results illustrate the gender disparity in safety performance of vehicles with same stars from 5-star ratings. The results from Table 4 show that the estimated parameter of vehicles with 5 stars from front-impact 5-star rating is fixed, which illustrated that the effect is stable and not affects by individual heterogeneity. The use of front-impact-5-stars vehicles could reduce the probability of severe injury of man drivers by 20.6% in front-impact crashes and that of woman drivers by 4.0%. However, the estimated parameter of vehicles with 4 stars from front-impact 5-star rating is significantly followed normal distribution (Figure 2a). Compared to vehicles with 3 or less stars from front-impact 5-star rating, using 4-stars vehicles makes the probability of severe injury for man drivers averagely decrease by 36.7%, for woman drivers averagely decrease only by 6.6%. Meanwhile, the mean of random distribution of the parameter of front-impact-4-stars vehicles is close to 0 for woman drivers, which also implied the individual heterogeneity is significantly related to safety performance of vehicles, especially for woman drivers.

For side-impact 5-star ratings of vehicles, using 5-stars vehicles has 64.9% and 36.6% lower probability of severe injury in driver-side-impact crashes respectively for man and woman drivers when comparing to vehicles with 4 or less stars. This result reflects that vehicles with 5 stars from side-impact 5-star rating can play additional protection performance to drivers in traffic crashes, but the protection performance of vehicles on woman drivers are still poorer than that of man drivers, and the gap is significant (Figure 2b). Considering the distribution characteristics of random parameters, 74.1% of man drivers have lower probability of severe injury in driver-side-impact crashes by using 5-stars vehicles from side-impact 5-star rating, and about 60.1% of woman drivers have lower probability of severe injury. It is illustrated that side-impact 5-star ratings of vehicles could relatively illustrate the safety performance of vehicles in traffic crashes for woman drivers, which could be explained by side-impact rating based on two tests that utilize woman dummy as rear-left passenger and drivers [1] (NHTSA, 2021). The side-impact 5-star rating pays more attention to the use of woman dummy in impact tests than other 5-star rating programs.

From the NHTSA, 5-star safety ratings of vehicles are identified by simulating the specific traffic crashes such as head-on crashes between two vehicles of a similar weight, then analyzing the injury severity of dummies that represent driver or passenger. In 5-star safety rating project, a man dummy could be as the representative of 50% of men in real-world society, but a woman dummy could only be as the representative of 5% of women. Some researchers found the risk of being injured or fatal for women in traffic crashes is significantly associated with their body shape, especially height [48,49]. Therefore, the woman dummy in 5-star safety rating project is not reasonable to be an effective representative of women in evaluating the safety performance of vehicles. It is explained why vehicles with 5-star safety ratings have significant heterogeneity in injury severity for drivers, and why have averagely 0% change of the probability of severe injury for woman drivers. Welsh et al., and Barry also focused on the dummies in vehicle impact safety testing, and they found woman dummies in testing represents the extremes of women involving real-world traffic crashes [50,51]. Therefore, it is essential to adding more suitable woman dummy to reduce the heterogeneity of safety performance of vehicles with the same 5-star ratings across woman drivers involving traffic crashes. Besides, the woman dummy in 5-star safety rating project is designed by scaling down the man dummy without considering the biomechanical disparity of gender. Bose et al., found woman drivers have higher risk of being chest and spine injured than man drivers when using rationally seat belt. And they proposed that the gender disparities in traffic safety are depended on injury tolerance and associate, biomechanical response [11]. The results also were found by Obeng; Ulfarsson and Mannering [8,19]. Therefore, the testing dummies in vehicle safety tests should reconsider the representativeness to change the man-oriented design.

Although the current study revealed multiple important findings, there are several limitations to the current study. First, the number of considered observed factors is limited. The speed difference of the two-vehicle at the time of crash, drivers’ physical characteristics (e.g., height and weight) and others should be considered. Second, vehicle safety is evaluated by the injury severity of dummies in vehicle impact testing, which mainly collected the impact data of head, chest, and femur. Lastly, this paper did not focus on the relationship between the injury severity of different body parts and vehicle ratings. Those limitations should be addressed in follow-up studies.

## 6. Conclusions

In this study, random parameter model was developed to identify relationship between vehicle safety ratings from 5-star rating and drivers’ injury severity by gender using real-world data from 2015–2020 two-vehicle crashes in Maryland, the United States, and models were controlled by drivers’ characteristics, vehicle, and environmental characteristics. This paper quantified the applicability of overall, front-impact and side-impact safety ratings of vehicles for drivers.

The dataset showed that more than 90% of vehicles involving two-vehicle crashes obtained 4 or 5 stars in the overall, front-impact and side-impact safety rating respectively, which implied that relatively lower safety ratings of vehicles had lost the practical significance of effectively distinguishing the safety performance of vehicles in traffic crashes. In addition, the study found that the 5-star ratings showed a significant effect on the probability of severe injury for drivers in two-vehicle crashes but is constrained by drivers’ gender. The results showed that, *ceteris paribus*, woman drivers were more likely to be seriously injured in crashes than man drivers in vehicles with the same safety ratings. Moreover, there was significant individual heterogeneity in the effect of vehicle with different safety ratings on driver injury severity. The using of vehicles with more stars from 5-star safety rating to some extent, can reduce the risk of being seriously injured for most or even 100% of man drivers. However, the probability of woman drivers being seriously injured was reduced by approximately 5% on average for vehicles with higher ratings in the overall and front-impact 5-star rating, and individual heterogeneity showed a difference of nearly 50% in positive and negative effects. The overall and front-impact 5-star ratings of vehicles could not provide reasonable information as the safety performance of vehicles in traffic crashes for woman drivers.

In conclusion, there was a significant gender difference in vehicles with different 5-star safety ratings, and the existing 5-star safety ratings is no longer applicable to explaining the protectiveness of vehicles in actual traffic environment, especially for woman drivers. Furthermore, man drivers are accounted for about 60% of all drivers in 1963 but the percentage has gradually decreased and was less than 50% in 2010 [52]. However, the 5-star safety ratings of vehicles are man-oriented and not suitable for woman drivers. Therefore, there is a serious gender inequality in the safety performance of vehicles for drivers, and it is reasonable and essential to pay more attention to women by improving the design of woman dummies in vehicle impact tests. The findings from this study call for gender equality in transportation system.

## Figures and Tables

**Table 1:** Descriptive statistics of the two-vehicle crash data.

Variable Category	Variable	Frequency	Percentage of Drivers Involving Severe Injury (%)
Driver characteristics	Severe injury	1364	100
	Non-severe injury	13,398	0
	Woman driver	7497	10.9
	Man driver	7283	7.5
	Younger driver (≤24 years)	2817	8.1
	Middle-age driver (25–59 years)	9996	9.3
	Older driver (≥60 years)	1949	10.6
	The driver’s residence is Maryland	2302	9.7
Driving characteristics	Driver was changing speed on the way	4951	7.5
	Opposite driver was at fault in two-vehicle crash	7062	11.2
Vehicle characteristics	Vehicle with overall 5 stars	9792	8.9
	Vehicle with overall 4 star	4536	9.8
	Vehicle with model age over 6 years old	1488	8.2
	Vehicle involving front-impact location	9041	9.7
	Vehicle involving driver-side-impact location	1196	11.4
Crash characteristics	Crash on dark-no light road	600	13.8
	Crash in raining/snowing/sleeting/mixing	1914	7.9
	Crash at a signal-control way	6550	10.8
	Crash occurred at Intersection	7810	10.5
	Crash on the way with speed limit 25–35 mph	7132	9.4
	Crash on the way with speed limit 40–55 mph	6001	10.3
	Crash on the way with speed limit over 60 mph(Interval is 5 mph)	583	5.0
	Crash on the road with holes/ruts/foreign material	1190	6.3
Interaction terms	Woman driver × Vehicle with overall 5 stars	4828	10.4
	Woman driver × Vehicle with overall 4 stars	2455	11.9

**Table 2:** Descriptive statistics of the key data including front-impact and side-impact crashes.

Variable Category	Variable	Front-Impact Crashes		Side-Impact Crashes	
		Frequency	Percentage of Drivers Involving Severe Injury (%)	Frequency	Percentage of Drivers involving Severe Injury (%)
Driver characteristics	Severe injury	978	100	81	100
	Non-severe injury	8063	0	682	0
	Woman driver	4541	11.2	371	13.2
	Man driver	4500	8.2	392	8.2
Interaction terms	Woman driver × Vehicle with front-impact or side-impact 5 stars	1650	10.5	322	13.7
	Woman driver × Vehicle with front-impact or side-impact 4 stars	2556	11.5	34	11.76

**Table 3:** Model results considering overall 5-star ratings of vehicles.

Variable	Estimated Parameter	S.E.	*p*-Value	Odds Ratio [95% CI]
Constant	−3.653 ***	0.211	<0.001	−
*s.d.* *of “Constant”*	1.969 ***	0.050	<0.001	−
Older driver (≥60 years)	0.148 **	0.070	0.035	1.160 [1.010,1.331]
The driver’s residence is Maryland	0.264 ***	0.076	<0.001	1.302 [1.122,1.510]
*s.d.* *of “The driver’s residence is Maryland”*	0.508 ***	0.038	<0.001	−
Driver was changing speed on the way	−0.256 ***	0.058	<0.001	0.774 [0.691,0.868]
Opposite driver was at fault in two−vehicle crash	0.525 ***	0.052	<0.001	1.690 [1.528,1.872]
Vehicle with overall 5 stars	−0.416 ***	0.146	0.004	0.660 [0.495,0.879]
*s.d.* *of “Vehicle with overall 5 stars”*	0.119 ***	0.044	0.007	−
Vehicle with overall 4 stars	−0.580 ***	0.159	<0.001	0.560 [0.410,0.764]
*s.d.* *of “Vehicle with overall 4stars”*	1.084 ***	0.068	<0.001	−
Vehicle with model age over 6 years old	−0.193 **	0.088	0.029	0.824 [0.694,0.980]
Vehicle involving front−impact location	0.172 ***	0.062	0.006	1.188 [1.052,1.342]
Vehicle involving driver−side−impact location	0.356 ***	0.095	<0.001	1.428 [1.185,1.719]
Crash on dark−no light road	0.620 ***	0.110	<0.001	1.859 [1.498,2.309]
Crash in raining/snowing/sleeting/mixing	−0.189 **	0.076	0.013	0.828 [0.712,0.961]
Crash at a signal−control way	−0.270 **	0.134	0.044	0.763 [0.586,0.993]
*s.d.* *of “Crash at a signal−control way”*	0.411 ***	0.050	<0.001	−
*heterogeneity in mean by “Crash occurred at Intersection”*	0.630 ***	0.135	<0.001	−
Crash on the way with speed limit 25−35 mph	0.556 ***	0.129	<0.001	1.744 [1.354,2.243]
Crash on the way with speed limit 40−55 mph	0.717 ***	0.129	<0.001	2.048 [1.590,2.640]
Crash on the way with speed limit over 60 mph	−0.944 ***	0.336	0.005	0.389 [0.201,0.752]
*s.d.* *of “Crash on the way with speed limit over 60 mph”*	2.412 ***	0.330	<0.001	−
Woman driver × Vehicle with overall 5 stars	0.390 ***	0.061	<0.001	1.477 [1.310,1.664]
Woman driver × Vehicle with overall 4 stars	0.579 ***	0.092	<0.001	1.784 [1.489,2.138]

Noted: ***, **, represent significance at 1%, 5% level.

**Table 4:** Model results considering front-impact and side-impact 5-star ratings of vehicles.

Variable Description	Front−Impact Crashes				Driver−Side−Impact Crashes			
	Parameter	S.E	*p*-Value	Odds Ratio [95% CI]	Parameter	S.E	*p*-Value	Odds Ratio [95% CI]
Constant	−3.108 ***	0.186	<0.001	−	−3.143 ***	0.565	<0.001	−
*s.d.* *of “Constant”*	1.465 ***	0.051	<0.001	−	0.969 ***	0.160	<0.001	−
The driver’s residence is Maryland	0.253 ***	0.090	0.005	1.288 [1.081,1.536]	−	−	−	−
Driver was changing speed on the way	−0.390 ***	0.077	<0.001	0.677 [0.582,0.787]	−	−	−	−
Opposite driver was at fault in two−vehicle crash	0.593 ***	0.059	<0.001	1.809 [1.613,2.030]	−	−	−	−
Vehicle involving front−impact location or side−impact 5 stars	−0.230 *	0.121	0.057	0.794 [0.628,1.007]	−1.146 ***	0.331	<0.001	0.318 [0.166,0.608]
*s.d.* *of “Vehicle involving front−impact location or side−impact 5 stars”*	−	−	−	−	1.774 ***	0.211	<0.001	−
Vehicle involving front−impact location or side−impact 4 stars	−0.457 ***	0.120	<0.001	0.633 [0.500,0.801]	−	−	−	−
*s.d.* *of “Vehicle involving front−impact location or side−impact 4 stars”*	0.754 ***	0.059	<0.001	−	−	−	−	−
Crash on dark−no light road	0.677 ***	0.125	<0.001	1.968 [1.540,2.514]	0.908 *	0.470	0.053	2.479 [0.988,6.228]
Crash in raining/snowing/sleeting/mixing	−0.190 **	0.088	0.031	0.827 [0.696,0.983]	−	−	−	−
Crash at a signal−control way	0.197 ***	0.058	<0.001	1.218 [1.086,1.365]	0.911 ***	0.224	<0.001	2.487 [1.602,3.857]
Crash on the way with speed limit 25−35 mph	0.608 ***	0.138	<0.001	1.837 [1.402,2.408]	1.044 **	0.505	0.039	2.840 [1.054,7.645]
Crash on the way with speed limit 40−55 mph	0.817 ***	0.139	<0.001	2.264 [1.725,2.971]	1.161 **	0.529	0.028	3.193 [1.131,9.007]
Woman driver × Vehicle with front−impact or side−impact 5 stars	0.189 **	0.092	0.040	1.208 [1.008,1.448]	0.690 ***	0.228	0.003	1.994 [0.166,0.608]
Woman driver × Vehicle with front−impact or side−impact 4 stars	0.389 ***	0.080	<0.001	1.476 [1.260,1.726]	−	−	−	−

Noted: ***, **, * represent significance at 1%, 5%, 10% level.

## Data Availability

Restrictions apply to the availability of these data. Data was obtained from the State of Maryland and are available https://opendata.maryland.gov/ (22 February 2022) with the permission of the State of Maryland.
